# Burnout in nursing: a theoretical review

**DOI:** 10.1186/s12960-020-00469-9

**Published:** 2020-06-05

**Authors:** Chiara Dall’Ora, Jane Ball, Maria Reinius, Peter Griffiths

**Affiliations:** 1 School of Health Sciences, and Applied Research Collaboration Wessex, Highfield Campus, University of Southampton, Southampton, SO17 1BJ UK; 2 Department of Learning, Informatics, Management and Ethics, Karolinska Institutet, Tomtebodavägen 18a, 17177 Solna, Sweden

**Keywords:** Burnout, Nursing, Maslach Burnout Inventory, Job demands, Practice environment

## Abstract

**Background:**

Workforce studies often identify burnout as a nursing ‘outcome’. Yet, burnout itself—what constitutes it, what factors contribute to its development, and what the wider consequences are for individuals, organisations, or their patients—is rarely made explicit. We aimed to provide a comprehensive summary of research that examines theorised relationships between burnout and other variables, in order to determine what is known (and not known) about the causes and consequences of burnout in nursing, and how this relates to theories of burnout.

**Methods:**

We searched MEDLINE, CINAHL, and PsycINFO. We included quantitative primary empirical studies (published in English) which examined associations between burnout and work-related factors in the nursing workforce.

**Results:**

Ninety-one papers were identified. The majority (*n* = 87) were cross-sectional studies; 39 studies used all three subscales of the Maslach Burnout Inventory (MBI) Scale to measure burnout. As hypothesised by Maslach, we identified high workload, value incongruence, low control over the job, low decision latitude, poor social climate/social support, and low rewards as predictors of burnout. Maslach suggested that turnover, sickness absence, and general health were effects of burnout; however, we identified relationships only with general health and sickness absence. Other factors that were classified as predictors of burnout in the nursing literature were low/inadequate nurse staffing levels, ≥ 12-h shifts, low schedule flexibility, time pressure, high job and psychological demands, low task variety, role conflict, low autonomy, negative nurse-physician relationship, poor supervisor/leader support, poor leadership, negative team relationship, and job insecurity. Among the outcomes of burnout, we found reduced job performance, poor quality of care, poor patient safety, adverse events, patient negative experience, medication errors, infections, patient falls, and intention to leave.

**Conclusions:**

The patterns identified by these studies consistently show that adverse job characteristics—high workload, low staffing levels, long shifts, and low control—are associated with burnout in nursing. The potential consequences for staff and patients are severe. The literature on burnout in nursing partly supports Maslach’s theory, but some areas are insufficiently tested, in particular, the association between burnout and turnover, and relationships were found for some MBI dimensions only.

## Introduction

The past decades have seen a growing research and policy interest around how work organisation characteristics impact upon different outcomes in nursing. Several studies and reviews have considered relationships between work organisation variables and outcomes such as quality of care, patient safety, sickness absence, turnover, and job dissatisfaction [1–4]. Burnout is often identified as a nursing ‘outcome’ in workforce studies that seek to understand the effect of context and ‘inputs’ on outcomes in health care environments. Yet, burnout itself—what constitutes it, what factors contribute to its development, and what the wider consequences are for individuals, organisations, or their patients—is not always elucidated in these studies.

The term *burnout* was introduced by Freudenberger in 1974 when he observed a loss of motivation and reduced commitment among volunteers at a mental health clinic [5]. It was Maslach who developed a scale, the Maslach Burnout Inventory (MBI), which internationally is the most widely used instrument to measure burnout [6]. According to Maslach’s conceptualisation, burnout is a response to excessive stress at work, which is characterised by feelings of being emotionally drained and lacking emotional resources—Emotional Exhaustion; by a negative and detached response to other people and loss of idealism—Depersonalisation; and by a decline in feelings of competence and performance at work—reduced Personal Accomplishment [7].

Maslach theorised that burnout is a state, which occurs as a result of a prolonged mismatch between a person and at least one of the following six dimensions of work [7–9]:

1. Workload: excessive workload and demands, so that recovery cannot be achieved.
2. Control: employees do not have sufficient control over the resources needed to complete or accomplish their job.
3. Reward: lack of adequate reward for the job done. Rewards can be financial, social, and intrinsic (i.e. the pride one may experience when doing a job).
4. Community: employees do not perceive a sense of positive connections with their colleagues and managers, leading to frustration and reducing the likelihood of social support.
5. Fairness: a person perceiving unfairness at the workplace, including inequity of workload and pay.
6. Values: employees feeling constrained by their job to act against their own values and their aspiration or when they experience conflicts between the organisation’s values.

Maslach theorised these six work characteristics as factors causing burnout and placed deterioration in employees’ health and job performance as outcomes arising from burnout [7].

Subsequent models of burnout differ from Maslach’s in one of two ways: they do not conceptualise burnout as an exclusively work-related syndrome; they view burnout as a process rather than a state [10].

The job resources-demands model [11] builds on the view of burnout as a work-based mismatch but differs from Maslach’s model in that it posits that burnout develops via two separate pathways: excessive job demands leading to exhaustion, and insufficient job resources leading to disengagement. Along with Maslach and Schaufeli, this model sees burnout as the negative pole of a continuum of employee’s well-being, with ‘work engagement’ as the positive pole [12].

Among those who regard burnout as a process, Cherniss used a longitudinal approach to investigate the development of burnout in early career human services workers. Burnout is presented as a process characterised by negative changes in attitudes and behaviours towards clients that occur over time, often associated with workers’ disillusionment about the ideals that had led them to the job [13]. Gustavsson and colleagues used this model in examining longitudinal data on early career nurses and found that exhaustion was a first phase in the burnout process, proceeding further only if nurses present dysfunctional coping (i.e. cynicism and disengagement) [14].

Shirom and colleagues suggested that burnout occurs when individuals exhaust their resources due to long-term exposures to emotionally demanding circumstances in both work and life settings, suggesting that burnout is not exclusively an occupational syndrome [15, 16].

This review aims to identify research that has examined theorised relationships with burnout, in order to determine what is known (and not known) about the factors associated with burnout in nursing and to determine the extent to which studies have been underpinned by, and/or have supported or refuted, theories of burnout.

## Methods

### Design

This was a theoretical review conducted according to the methodology outlined by Campbell et al. and Pare et al. [17, 18]. Theoretical reviews draw on empirical studies to understand a concept from a theoretical perspective and highlight knowledge gaps. Theoretical reviews are systematic in terms of searching and inclusion/exclusion criteria and do not include a formal appraisal of quality. They have been previously used in nursing, but not focussing on burnout [19]. While no reporting guideline for theoretical reviews currently exists, the PRISMA-ScR was deemed to be suitable, with some modifications, to enhance the transparency of reporting for the purposes of this review. The checklist, which can be found as Additional file 2, has been modified as follows:

- Checklist title has been modified to indicate that the checklist has been adapted for theoretical reviews.
- Introduction (item 3) has been modified to reflect that the review questions lend themselves to a theoretical review approach.
- Selection of sources of evidence (item 9) has been modified to state the process for selecting sources of evidence in the theoretical review.
- Limitations (item 20) has been amended to discuss the limitations of the theoretical review process.
- Funding (item 22) has been amended to describe sources of funding and the role of funders in the theoretical review.

All changes from the original version have been highlighted.

### Literature search

A systematic search of empirical studies examining burnout in nursing published in journal articles since 1975 was performed in May 2019, using MEDLINE, CINAHL, and PsycINFO. The main search terms were ‘burnout’ and ‘nursing’, using both free-search terms and indexed terms, synonyms, and abbreviations. The full search and the total number of papers identified are in Additional file 1.

We included papers written in English that measured the association between burnout and work-related factors or outcomes in all types of nurses or nursing assistants working in a healthcare setting, including hospitals, care homes, primary care, the community, and ambulance services. Because there are different theories of burnout, we did not restrict the definition of burnout according to any specific theory. Burnout is a work-related phenomenon [8], so we excluded studies focussing exclusively on personal factors (e.g. gender, age). Our aim was to identify theorised relationships; therefore, we excluded studies which were only comparing the levels of burnout among different settings (e.g. in cancer services vs emergency departments). We excluded literature reviews, commentaries, and editorials.

### Data extraction and quality appraisal

**Fig. 1:**
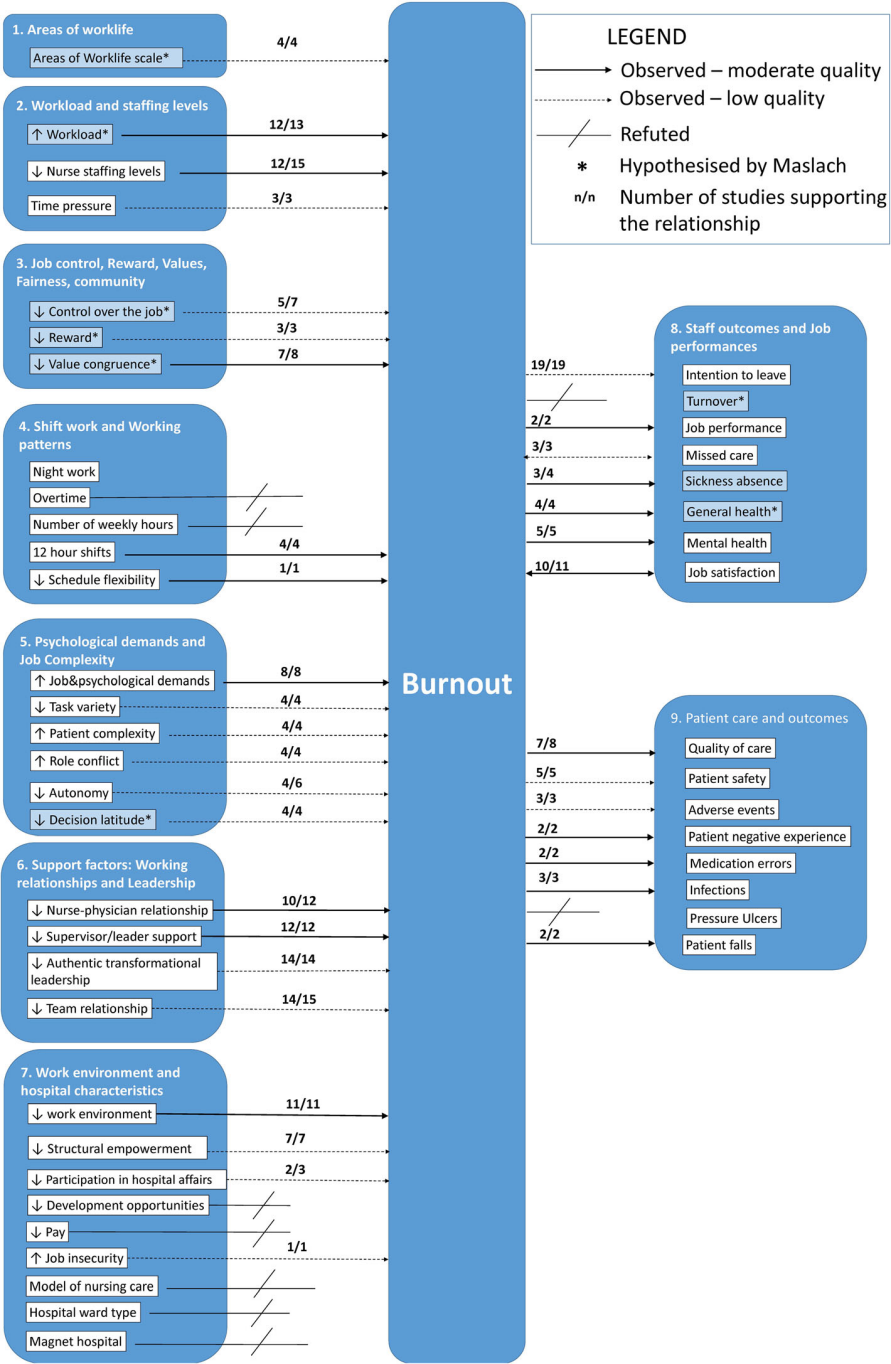
Graphical representation of strength of relationships with burnout

The following data were extracted from included studies: country, setting, sample size, staff group, measure of burnout, variables the relationship with burnout was tested against, and findings against the hypothesised relationships. One reviewer (MEB) extracted data from all the studies, with CDO and JEB extracting 10 studies each to check for agreement in data extraction. In line with the theoretical review methodology, we did not formally assess the quality of studies [19]. However, in Additional file 3, we have summarised the key aspects of quality for each study, covering generalisability (e.g. a multisite study with more than 500 participants); risk of bias from common methods variance (e.g. burnout and correlates assessed with the same survey. This bias arises when there is a shared (common) variance because of the common method rather than a true (causal) association between variables); evidence of clustering (e.g. nurses nested in wards, wards nested in hospitals); and evidence of statistical adjustment (e.g. the association between burnout and correlates has been adjusted to control for potentially influencing variables). It should be noted that cells are shaded in green when the above-mentioned quality standards have been met, and in red when they have not. In the ‘Discussion’ section, we offer a reflection on the common limitations of research in the field and present a graphic summary of the ‘strength of evidence’ in Fig. 1.

### Data synthesis

Due to the breadth of the evidence, we summarised extracted data by identifying common categories through a coding frame. The starting point of the coding frame was the burnout multidimensional theory outlined by Maslach [7]. We then considered whether the studies’ variables fit into Maslach’s categorisation, and where they did not, we created new categories. We identified nine broad categories: (1) Areas of Worklife; (2) Workload and Staffing Levels; (3) Job Control, Reward, Values, Fairness, and Community; (4) Shift Work and Working Patterns; (5) Psychological Demands and Job Complexity; (6) Support Factors: Working Relationships and Leadership; (7) Work Environment and Hospital Characteristics; (8) Staff Outcomes and Job Performance; and (9) Patient Care and Outcomes. In the literature, categories 1–7 were treated as predictors of burnout and categories 8 and 9 as outcomes, with the exception of missed care and job satisfaction which were treated both as predictors and outcomes.

When the coding frame was finalised, CDO and MLR applied it to all studies. Where there was disagreement, a third reviewer (JEB) made the final decision.

## Results

**Fig. 2:**
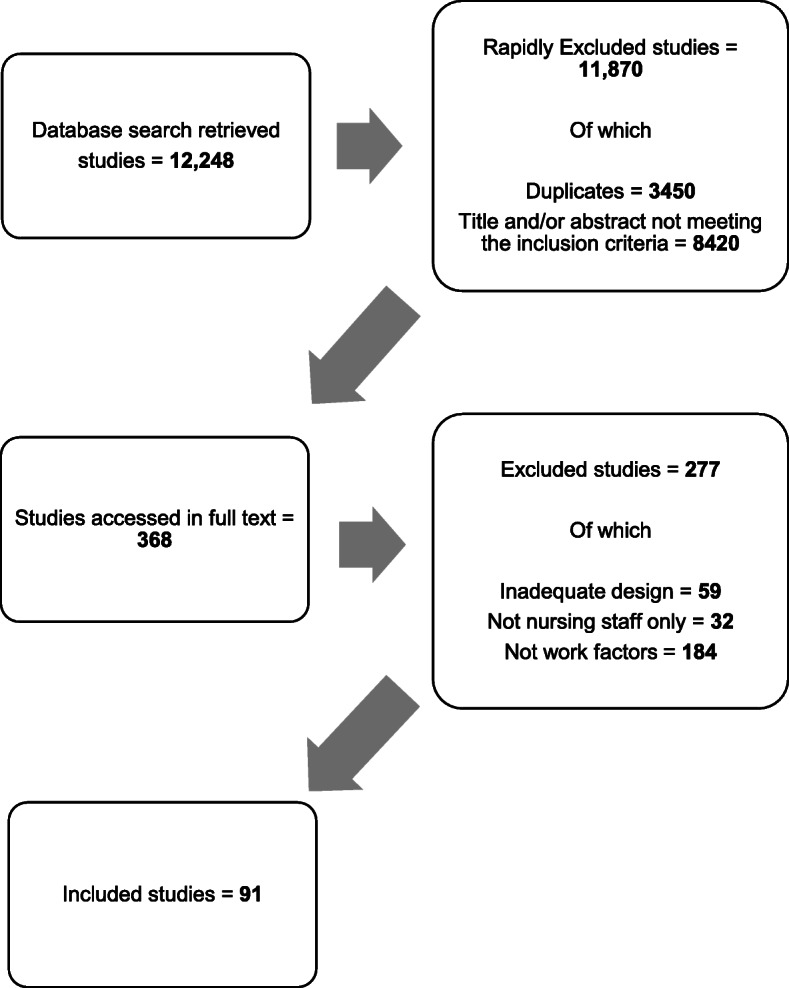
Study selection flow chart

The database search yielded 12 248 studies, of which 11 870 were rapidly excluded as either duplicates or titles and/or abstract not meeting the inclusion criteria. Of the 368 studies accessed in full text, 277 were excluded, and 91 studies were included in the review. Figure 2 presents a flow chart of the study selection.

The 91 studies identified covered 28 countries; four studies included multiple countries, and in one, the country was not reported. Most were from North America (*n* = 35), Europe (*n* = 28), and Asia (*n* = 18).

The majority had cross-sectional designs (*n* = 87, 97%); of these, 84 were entirely survey-based. Three studies were longitudinal. Most studies were undertaken in hospitals (*n* = 82). Eight studies surveyed nurses at a national level, regardless of their work setting.

Sample sizes ranged from hundreds of hospitals (max = 927) with hundreds of thousands of nurses (max = 326 750) [20] to small single-site studies with the smallest sample being 73 nurses [21] (see Additional file 3).

The relationships examined are summarised in Table 1.

**Table 1:** Summary of studies’ results

	Hypothesised by Maslach’s theory	Observed	Refuted**	Number of studies supporting the relationship
1. Areas of worklife				
Areas of worklife (high score on Areas of Worklife Scale)	√	√		4 out of 4
2. Workload and staffing levels				
High workload	√	√* (definitive for EE only)		12 out of 13
Nurse staffing levels (low/inadequate)		√*		12 out of 15
Time pressure		√* (definitive for EE only)		3 out of 3
3. Job control, reward, values, fairness, community				
Low control over the job	√	√*		5 out of 7
Low reward	√	√*		3 out of 3
Low value congruence	√	√* (definitive for EE and DEP)		7 out of 8
4. Shift work and working patterns				
Night work			√	
Overtime			√	
Number of hours worked per week			√	
≥ 12-h shifts		√*(definitive for EE only)		4 out of 4
Low schedule flexibility		√* (definitive only for EE)		1 out of 1
5. Demands and job complexity				
Job and psychological demands		√* (definitive for EE only)		8 out of 8
Low task variety		√*		4 out of 4
High patient complexity		√*		4 out of 4
Role conflict		√* (definitive for EE only)		4 out of 4
Low autonomy		√*		4 out of 6
Low decision latitude	√	√*		4 out of 4
6. Support factors: working relationships and leadership				
Negative nurse-physician relationship		√*		10 out of 12
Low supervisor/leader support		√*		12 out of 12
Leadership styles that are not authentic and transformational		√* (definitive only for EE)		14 out of 14
Negative team relationship		√*		14 out of 15
7. Work environment and hospital characteristics				
Negative work environment (global scale)		√* (definitive for EE only)		11 out of 11
Low Structural/organisation empowerment		√* (definitive for EE only)		7 out of 7
Limited Participation in hospital affairs (including policy and research)		√*		2 out of 3
No development opportunities			√	
Low pay			√	
High job insecurity		√*		1 out of 1
Model of nursing care			√	
Specialised hospital/ward type			√	
Magnet hospital			√	
8. Staff outcomes and job performance				
Intention to leave		√*		19 out of 19
Turnover	√		√	
Low job performance		√*		2 out of 2
Missed care		√***		3 out of 3
Sickness absence	√	√*		3 out of 4
Poor general health	√	√* (definitive for EE only)		4 out of 4
Mental health issues (including depression)		√*		5 out of 5
Job dissatisfaction		√***		10 out of 11
9. Patient care and outcomes				
Poor quality of care		√*		7 out of 8
Poor patient safety		√*		5 out of 5
Adverse events		√*		3 out of 3
Patient negative experience (including dissatisfaction and verbal abuse)		√*		2 out of 2
Medication errors		√*		2 out of 2
Infections		√*		3 out of 3
Pressure ulcers			√	
Patient falls		√*		2 out of 2

*Partial evidence (e.g. relationship established with some but not all burnout subscales)

**Refuted when there is consistent evidence that a hypothesised relationship does not exist (e.g. large studies with no confidence intervals shown if no association)

***Observed in multiple directions

### Measures of burnout

Most studies used the Maslach Burnout Inventory Scale (*n* = 81), which comprises three subscales reflecting the theoretical model: Emotional Exhaustion, Depersonalisation, and reduced Personal Accomplishment. However, less than half (47%, *n* = 39) of the papers measured and reported results with all three subscales. Twenty-three papers used the Emotional Exhaustion subscale only, and 11 papers used the Emotional Exhaustion and Depersonalisation subscales. In nine studies, the three MBI subscales were summed up to provide a composite score of burnout, despite Maslach and colleagues advising against such an approach [22].

Five studies used the Copenhagen Burnout Inventory (CBI) [23]. This scale consists of three dimensions of burnout: personal, work-related, and client-related. Two studies used the Malach-Pines Scale [24], and one used the burnout subscale of the Professional Quality of Life Measure (ProQoL5) scale, which posits burnout as an element of compassion fatigue [25]. Two studies used idiosyncratic measures of burnout based on items from other instruments [20, 26].

### Factors examined in relation to burnout: an overview

The studies which tested the relationships between burnout and Maslach’s six areas of worklife—workload, control, reward, community, fairness, and values—typically supported Maslach’s theory that these areas are predictors of burnout. However, some evidence is based only on certain MBI dimensions. High scores on the *Areas of Worklife Scale* [27] (indicating a higher degree of congruence between the job and the respondent) were associated with less likelihood of burnout, either directly [28, 29] or through high occupational coping self-efficacy [30] and presence of civility norms and co-worker incivility [31].

The majority of studies looking at job characteristics hypothesised by the Maslach model considered workload (*n* = 31) and job control and reward (*n* = 10). While only a few studies (*n* = 9) explicitly examined the hypothesised relationships between burnout and community, fairness, or values, we identified 39 studies that covered ‘supportive factors’ including relationships with colleagues and leadership.

A large number of studies included factors that fall outside of the Maslach model. Six main areas were identified:

- Working patterns and shifts working (*n* = 15)
- Features inherent in the job such as psychological demand and complexity (*n* = 24)
- Job support from working relationships and leadership (*n* = 39)
- Hospital or environmental characteristics (*n* = 28)
- Staff outcomes and job performance (*n* = 33)
- Patient outcomes (*n* = 17)
- Individual attributes (personal or professional) (*n* = 16)

### Workload and staffing levels

Workload and characteristics of jobs that contribute to workload, such as staffing levels, were the most frequently examined factor in relation to burnout. Thirty studies found an association between high workload and burnout.

Of these, 13 studies looked specifically at measures of workload as a predictor of burnout. Workload was associated with Emotional Exhaustion in five studies [32–36], with some studies also reporting a relationship with Depersonalisation, and others Cynicism. Janssen reported that ‘mental work overload’ predicted Emotional Exhaustion [37]. Three studies concluded that workload is associated with both Emotional Exhaustion and Depersonalisation [38–40]. Kitaoka-Higashiguchi tested a model of burnout and found that heavy workload predicted Emotional Exhaustion, which in turn predicted Cynicism [41]. This was also observed in a larger study by Greengrass et al. who found that high workload was associated with Emotional Exhaustion, which consequently predicted Cynicism [42]. One study reported no association between workload and burnout components [43], and one study found an association between manageable workload and a composite burnout score [44].

Further 15 studies looked specifically at nurse staffing levels, and most reported that when nurses were caring for a higher number of patients or were reporting staffing inadequacy, they were more likely to experience burnout. No studies found an association between better staffing levels and burnout.

While three studies did not find a significant association with staffing levels [32, 45, 46], three studies found that higher patient-to-nurse ratios were associated with Emotional Exhaustion [47–49], and in one study, higher patient-to-nurse-ratios were associated with Emotional Exhaustion, Depersonalisation, and Personal Accomplishment [50]. One study concluded that Emotional Exhaustion mediated the relationship between patient-to-nurse ratios and patient safety [51]. Akman and colleagues found that the lower the number of patients nurses were responsible for, the lower the burnout composite score [52]. Similar results were highlighted by Faller and colleagues [53]. Lower RN hours per patient day were associated with burnout in a study by Thompson [20].

When newly qualified RNs reported being short-staffed, they were more likely to report Emotional Exhaustion and Cynicism 1 year later [54]. In a further study, low staffing adequacy was associated with Emotional Exhaustion [55]. Similarly, Leineweber and colleagues found that poor staff adequacy was associated with Emotional Exhaustion, Depersonalisation, and Personal Accomplishment [56]. Leiter and Spence Laschinger explored the relationship between staffing adequacy and all MBI subscales and found that Emotional Exhaustion mediated the relationship between staffing adequacy and Depersonalisation [57]. Time pressure was investigated in three studies, which all concluded that reported time pressure was associated with Emotional Exhaustion [58–60].

In summary, there is evidence that high workload is associated with Emotional Exhaustion, nurse staffing levels are associated with burnout, and time pressure is associated with Emotional Exhaustion.

### Job control, reward, values, fairness, and community

Having control over the job was examined in seven studies. Galletta et al. found that low job control was associated with all MBI subscales [40], as did Gandi et al. [61]. Leiter and Maslach found that control predicted fairness, reward, and community, and in turn, fairness predicted values, and values predicted all MBI subscales [35]. Low control predicted Emotional Exhaustion only for nurses working the day shift [62], and Emotional Exhaustion was significantly related to control over practice setting [63]; two studies reported no effect of job control on burnout [44, 64].

Reward predicted Cynicism [35] and burnout on a composite score [44]. Shamian and colleagues found that a higher score in the effort and reward imbalance scale was associated with Emotional Exhaustion, and higher scores in the effort and reward imbalance scale were associated with burnout measured by the CBI [65].

Value congruence refers to a match between the requirements of the job and people’s personal principles [7]. Value conflicts were related with a composite score of burnout [44], and one study concluded that nurses with a high value congruence reported lower Emotional Exhaustion than those with a low value congruence, and nurses with a low value congruence experienced more severe Depersonalisation than nurses with a high value congruence [66]. Low value congruence was a predictor of all three MBI dimensions [35] and of burnout measured with the Malach-Pines Burnout Scale [67]. Two studies considered social capital, defined as a social structure that benefits its members including trust, reciprocity, and a set of shared values, and they both concluded that lower social capital in the hospital-predicted Emotional Exhaustion [33, 36]. A single study showed fairness predicted values, which in turn predicted all MBI Scales [35]. Two studies looked at community, and one found that community predicts a composite score of burnout [44], while the other found no relationships [35].

While not directly expressed in the terms described by Maslach, other studies demonstrate associations with possible causal factors, many of which are reflected in Maslach’s theory.

In summary, there is evidence that control over the job is associated with reduced burnout, and value congruence is associated with reduced Emotional Exhaustion and Depersonalisation.

### Working patterns and shift work

Shift work and working patterns variables were considered by 15 studies. Overall, there was mixed evidence on the relationship between night work, number of hours worked per week, and burnout, with more conclusive results regarding the association between long shifts and burnout, and the potential protective effect of schedule flexibility.

Working night shifts was associated with burnout (composite score) [68] and Emotional Exhaustion [62], but the relationship was not significant in two studies [69, 70]. Working on permanent as opposed to rotating shift patterns did not impact burnout [71], but working irregular shifts did impact a composite burnout score [72]. When nurses reported working a higher number of shifts, they were more likely to report higher burnout composite scores [68], but results did not generalise in a further study [69]. One study found working that overtime was associated with composite MBI score [73]. On-call requirement was not significantly associated with any MBI dimensions [71].

The number of hours worked per week was not a significant predictor of burnout according to two studies [25, 53], but having a higher number of weekly hours was associated with Emotional Exhaustion and Depersonalisation in one study [70]. Long shifts of 12 h or more were associated with all MBI subscales [74] and with Emotional Exhaustion [49, 75]. A study using the ProQoL5 burnout scale found that shorter shifts were protective of burnout [25].

Having higher schedule flexibility was protective of Emotional Exhaustion [46], and so was the ability to schedule days off for a burnout composite score [76]. Having more than 8 days off per month was associated with lower burnout [69]. Stone et al. found that a positive scheduling climate was protective of Emotional Exhaustion only [77].

In summary, we found an association between ≥ 12-h shifts and Emotional Exhaustion and between schedule flexibility and reduced Emotional Exhaustion.

### Psychological demands and job complexity

There is evidence from 24 studies that job demands and aspects intrinsic to the job, including role conflict, autonomy, and task variety, are associated with some burnout dimensions.

Eight studies considered psychological demands. The higher the psychological demands, the higher the likelihood of experiencing all burnout dimensions [72], and high psychological demands were associated with higher odds of Emotional Exhaustion [62, 78]. Emotional demands, in terms of hindrances, had an effect on burnout [67]. One study reported that job demands, measured with the Effort-Reward Imbalance Questionnaire, were correlated with all burnout dimensions [79], and similarly, Garcia-Sierra et al. found that demands predict burnout, measured with a composite scale of Emotional Exhaustion and Cynicism [80]. According to one study, job demands were not associated with burnout [73], and Rouxel et al. concluded that the higher the job demands, the higher the impact on both Emotional Exhaustion and Depersonalisation [64].

Four studies looked at task nature and variety, quality of job content, in terms of skill variety, skill discretion, task identity, task significance, influenced Emotional Exhaustion through intrinsic work motivation [37]. Skill variety and task significance were related to Emotional Exhaustion; task significance was also related to Personal Accomplishment [60]. Having no administrative tasks in the job was associated with a reduced likelihood to experience Depersonalisation [71]. Higher task clarity was associated with reduced levels of Emotional Exhaustion and increased Personal Accomplishment [58].

Patient characteristics/requirements were investigated in four papers. When nurses were caring for suffering patients and patients who had multiple requirements, they were more likely to experience Emotional Exhaustion and Cynicism. Similarly, caring for a dying patient and having a high number of decisions to forego life-sustaining treatments were associated with a higher likelihood of burnout (measured with a composite score) [76]. Stress resulting from patient care was associated with a composite burnout score [73]. Patient violence also had an impact on burnout, measured with CBI [81], as did conflict with patients [76].

Role conflict is a situation in which contradictory, competing, or incompatible expectations are placed on an individual by two or more roles held at the same time. Role conflict predicted Emotional Exhaustion [41], and so it did in a study by Konstantinou et al., who found that role conflict was associated with Emotional Exhaustion and Depersonalisation [34]; Levert and colleagues reported that role conflict correlated with Emotional Exhaustion, Depersonalisation, and Personal Accomplishment. They also considered role ambiguity, which correlated with Emotional Exhaustion and Depersonalisation, but not Personal Accomplishment [39]. Andela et al. investigated the impact of emotional dissonance, defined as the mismatch between the emotions that are felt and the emotions required to be displayed by organisations. They reported that emotional dissonance is a mediator between job aspects (i.e. workload, patient characteristics, and team issues) and Emotional Exhaustion and Cynicism. Rouxel et al. found that perceived negative display rules were associated with Emotional Exhaustion [64].

Autonomy related to Emotional Exhaustion and Depersonalisation [60], and in another study, it only related to Depersonalisation [43]. Low autonomy impacted Emotional Exhaustion via organisational trust [82]. Autonomy correlated with burnout [67]. There was no effect of autonomy on burnout according to two studies [58, 63]. Low decision-making at the ward level was associated with all MBI subscales [77]. Decision latitude impacted Personal Accomplishment only [36], and in one study, it was found to be related to Emotional Exhaustion [78]. High decision latitude was associated with Personal Accomplishment [41] and low Emotional Exhaustion [33].

Overall, high job and psychological demands were associated with Emotional Exhaustion, as was role conflict. Patient complexity was associated with burnout, while task variety, autonomy, and decision latitude were protective of burnout.

### Working relationships and leadership

Overall, evidence from 39 studies supports that having positive support factors and working relationships in place, including positive relationships with physicians, support from the leader, positive leadership style, and teamwork, might play a protective role towards burnout.

The quality of the relationship with physicians was investigated by 12 studies. In two studies, having negative relationships with physicians was associated with all MBI dimensions [77, 83]; quality of nurse-physician relationship was associated with Emotional Exhaustion and Depersonalisation, but not PA [50]. Two studies found an association with Emotional Exhaustion only [55, 84], and one concluded that quality of relationship with physicians indirectly supported PA [36]. This was also found by Leiter and Laschinger, who found that positive nurse-physician collaborations predicted Personal Accomplishment [57, 85]. When burnout was measured with composite scores of MBI and a not validated scale, two studies reported an association with nurse-physician relationship [20, 76], and two studies found no associations [56, 63].

Having support from the supervisor or leader was considered in 12 studies, which found relationships with different MBI dimensions. A relationship between low support from nurse managers and all MBI subscales was observed in one study [77], while two studies reported it is a protective factor from Emotional Exhaustion only [58, 83], and one that it was also associated with Depersonalisation [86]. Kitaoka-Higashiguchi reported an association only with Cynicism [41], and Jansen et al. found it was only associated with Depersonalisation and Personal Accomplishment [60]. Van Bogaert and colleagues found that support from managers predicted low Emotional Exhaustion and high Personal Accomplishment [84], but in a later study, it only predicted high Personal Accomplishment [36]. Regarding the relationship with the manager, it had a direct effect on Depersonalisation, and it moderated the effect of time pressure on Emotional Exhaustion and Depersonalisation [59]; a protective effect of a quality relationship with the head nurse on a composite burnout score was also reported [76]. Two studies using different burnout scales found an association between manager support and reduced burnout [25, 67]. Low trust in the leader showed a negative impact on burnout, measured with a composite score [87]. Two further studies focused on the perceived nurse manager’s ability: authors found that it was related to Emotional Exhaustion [46], and Emotional Exhaustion and Personal Accomplishment [50].

Fourteen studies looked at the leadership style and found that it affects burnout through different pathways and mechanisms. Boamah et al. found that authentic leadership—described as leaders who have high self-awareness, balanced processing, an internalised moral perspective, and transparency—predicted higher empowerment, which in turn predicted lower levels of Emotional Exhaustion and Cynicism a year later [54]. Authentic leadership had a negative direct effect on workplace bullying, which in turn had a direct positive effect on Emotional Exhaustion [88]. Effective leadership predicted staffing adequacy, which in turn predicted Emotional Exhaustion [57, 85]. Authentic leadership predicted all areas of worklife, which in turn predicted all MBI dimensions of burnout [30], and a similar pathway was identified by Laschiner and Read, although authentic leadership impacted Emotional Exhaustion only and it was also through civility norms and co-worker incivility [31]. Emotional Exhaustion mediated the relationship between authentic leadership and intention to leave the job [89]. ‘Leader empowering behaviour’ had an indirect effect on Emotional Exhaustion through structural empowerment [29], and empowering leadership predicted trust in the leader, which in turn was associated with burnout composite score [87]. Active management-by-exception was beneficial for Depersonalisation and Personal Accomplishment, passive laissez-faire leadership negatively affected Emotional Exhaustion and Personal Accomplishment, and rewarding transformational leadership protected from Depersonalisation [90]. Contrary to this, Madathil et al. found that transformational leadership protected against Emotional Exhaustion, but not Depersonalisation, and promoted Personal Accomplishment [43]. Transformational leadership predicted positive work environments, which in turn predicted lower burnout (composite score) [44]. Positive leadership affected Emotional Exhaustion and Depersonalisation [56] and burnout measured with a non-validated scale [20].

Teamwork and social support were also explored. Co-worker cohesion was only related to Depersonalisation [58]; team collaboration problems predicted negative scores on all MBI subscales [38], and workplace support protected from Emotional Exhaustion [72]. Similarly, support received from peers had a protective effect on Emotional Exhaustion [60]. Collegial support was related to Emotional Exhaustion and Personal Accomplishment [39], and colleague support protected from burnout [67]. Interpersonal conflict affected Emotional Exhaustion through role conflict, but co-worker support had no effect on any burnout dimensions [41], and similarly, co-worker incivility predicted Emotional Exhaustion [31], and so did bullying [88]. Poor team communication was associated with all MBI dimensions [40], staff issues predicted burnout measured with a composite score [73], and so did verbal violence from colleagues [68]. One study found that seeking social support was not associated with any of the burnout dimensions, while another study found that low social support predicted Emotional Exhaustion [37], and social support was associated with lower Emotional Exhaustion and higher Personal Accomplishment [21]. Vidotti et al. found an association between low social support and all MBI dimensions [62].

### Work environment and hospital characteristics

Eleven studies were considering the work environment measured with the PES-NWI scale [91], where higher scores indicate positive work environments. Five studies comprising diverse samples and settings concluded that the better rated the work environment, the lower the likelihood of experiencing Emotional Exhaustion [32, 47, 49, 51, 92], and four studies found the same relationship, but on both Emotional Exhaustion and Depersonalisation [50, 66, 93, 94]; only one study concluded there is an association between work environment and all MBI dimensions [95]. Negative work environments affected burnout (measured with a composite score) via job dissatisfaction [96]. One study looked at organisational characteristics on a single scale and found that a higher rating of organisational characteristics predicted lower Emotional Exhaustion [82]. Environmental uncertainty was related to all MBI dimensions [86].

Structural empowerment was also considered in relation to burnout: high structural empowerment led to lower Emotional Exhaustion and Cynicism via staffing levels and worklife interference [54]; in a study using a similar methodology, structural empowerment affected Emotional Exhaustion via Areas of Worklife [29]. The relationship between Emotional Exhaustion and Cynicism was moderated by organisational empowerment [40], and organisational support had a protective effect on burnout [67]. Hospital management and organisational support had a direct effect on Emotional Exhaustion and Personal Accomplishment [84]. Trust in the organisation predicted lower levels of Emotional Exhaustion [82] and of burnout measured with a composite MBI score [87].

Three studies considered whether policy involvement had an effect on burnout. Two studies on the same sample found that having the opportunity to participate in policy decisions was associated with reduced burnout (all subscales) [57, 85], and one study did not report results for the association [20]. Emotional Exhaustion mediated the relationship between nurses’ participation in hospital affairs and their intention to leave the job [97]; a further study did not found an association between participation in hospital affairs and Emotional Exhaustion, but only with Personal Accomplishment [50]. Lastly, one study investigated participation in research groups and concluded it was associated with reduced burnout measured with a composite score [76].

There was an association between opportunity for career advancement and all MBI dimensions [77]; however, another study found that having promotion opportunities was not related to burnout [79]. Moloney et al. found that professional development was not related to burnout [67]. Two studies considered pay. In one study, no effect was found on any MBI dimension [73], and a very small study (*n* = 78 nurses) reported an effect of satisfaction with pay on Emotional Exhaustion and Depersonalisation [34]. Job insecurity predicted Depersonalisation and PA [79].

When the hospital adopted nursing models of care rather than medical models of care, nurses were more likely to report high levels of Personal Accomplishment [57, 85]. However, another study found no significant relationship [20]. Regarding ward and hospital type, Aiken and Sloane found that RNs working in specialised AIDS units reported lower levels of Emotional Exhaustion [98]; however, ward type was not found to be significantly associated with burnout in a study on temporary nurses [53]. Working in different ward settings was not associated with burnout, but working in hospitals as opposed to in primary care was associated with lower Emotional Exhaustion [71]. Working in a small hospital was associated with a lower likelihood of Emotional Exhaustion, when compared to working in a community hospital [63]. Faller’s study also concluded that working in California was a significant predictor of reduced burnout.

When the hospitals’ investment in the quality of care was considered, one study found that having foundations for quality of care was associated with reduced Emotional Exhaustion only [50], but in another study, foundations for quality of care were associated with all MBI dimensions [83]. Working in a Magnet hospital was not associated with burnout [53].

In summary, having a positive work environment (generally work environments scoring higher on the PES-NWI scale) was associated with reduced Emotional Exhaustion, and so was higher structural empowerment. However, none of the organisational characteristics at the hospital level was consistently associated with burnout.

### Staff outcomes and job performance

Nineteen studies considered the impact of burnout on intention to leave. Two studies found that Emotional Exhaustion and Cynicism had a direct effect on turnover intentions [28, 99], and four studies reported that only Emotional Exhaustion affected intentions to leave the job [21, 32, 37, 100], with one of these indicating that Emotional Exhaustion affected also intention to leave the organisation [32], but one study did not replicate such findings [101] and concluded that only Cynicism was associated with intention to leave the job and nursing. Similarly, one study found that Cynicism was directly related to intention to leave [35]. A further study found that Emotional Exhaustion affected turnover intentions via job satisfaction [88], and one article reported that Emotional Exhaustion mediated the effect of authentic leadership on intention to leave [89]. Emotional Exhaustion was a mediator between nurses’ involvement with decisions and intention to leave the organisation [97]. Burnout measured on a composite score was associated with a higher intention to leave [96]. Laeeque et al. reported that burnout, captured with CBI, related to intention to leave [81]; Estryn-Behar et al. used the same scale to measure burnout and found that high burnout was associated with higher intention to leave in all countries, except for Slovakia [102]. Burnout, measured with the Malach-Pines Scale, was associated with intention to quit, and stronger associations were found for nurses who had higher perceptions of organisational politics [103]. Burnout (Malach-Pines Scale) predicted both the intention to leave the job and nursing [67]. Three studies investigated the relationship between burnout and intention to leave; one of these aggregated all job outcomes in a single variable (i.e. job satisfaction, intention to leave the hospital, applied for another job, and intention to leave nursing) and reported that Depersonalisation and Personal Accomplishment predict job outcomes [84]; they replicated a similar approach and found the same associations [36]. They later found that all MBI dimensions were associated with leaving the nursing profession [104]. Only one study in a sample of 106 nurses from one hospital found an association between Depersonalisation and turnover within 2 years [105].

Two studies looked at the effect of burnout on job performance: one found a negative association between burnout (measured with CBI) and both task performance and contextual performance [106]. Only Emotional Exhaustion was associated with self-rated and supervisor-rated job performance of 73 RNs [21]. Missed care was investigated in three studies, and it was found to be both predictor of Emotional Exhaustion [32], an outcome of burnout [20, 103].

Four studies considered sickness absence. When RNs had high levels of Emotional Exhaustion, they were more likely to experience short-term sickness absence (i.e. 1–10 days of absence), which was obtained from hospital administrative records. Similarly, Emotional Exhaustion was associated with seven or more days of absence in a longitudinal study [105]. Emotional Exhaustion was significantly associated with reported mental health absenteeism, but not reported physical health absenteeism, and sickness absence from administrative records [21]. One study did not find any meaningful relationships between burnout and absenteeism [107].

Emotional Exhaustion was a significant predictor of general health [73], and in a further study, both Emotional Exhaustion and Personal Accomplishment were associated with perceived health [70]. Final-year nursing students who experienced health issues were more likely to develop high burnout when entering the profession [26]. When quality of sleep was treated both as a predictor and outcome of burnout, relationships were found in both instances [106].

Focussing on mental health, one study found that burnout predicted mental health problems for newly qualified nurses [30], and Emotional Exhaustion and Cynicism predicted somatisation [42]. Depressive symptoms were predictive of Emotional Exhaustion and Depersonalisation, considering therefore depression as a predictor of burnout [108]. Rudman and Gustavsson also found that having depressive mood and depressive episodes were common features of newly qualified nurses who developed or got worse levels of burnout throughout their first years in the profession [26]. Tourigny et al. considered depression as a predictor and found it was significantly related to Emotional Exhaustion [107].

Eleven studies considered job satisfaction: of these, three treated job satisfaction as a predictor of burnout and concluded that higher levels of job satisfaction were associated with a lower level of composite burnout scores [52, 96] and all MBI dimensions [94]. According to two studies, Emotional Exhaustion and Cynicism predicted job dissatisfaction [54, 101], while four studies reported that Emotional Exhaustion only was associated with increased odds to report job dissatisfaction [73, 82, 88, 100]; one study reported that Cynicism only was associated with job dissatisfaction [99]. Rouxel et al. did not find support in their hypothesised model that Emotional Exhaustion and Depersonalisation predicted job satisfaction [64].

In summary, considering 39 studies, there is conflicting evidence on the direction of the relationship between burnout and missed care, mental health, and job satisfaction. An association between burnout and intention to leave was found, although only one small study reported an association between burnout and turnover. A moderate relationship was found for the effect of burnout on sickness absence, job performance, and general health.

### Patient care and outcomes

Among the patient outcomes of burnout, quality of care was investigated by eight studies. Two studies in diverse samples and settings reported that high Emotional Exhaustion, high Depersonalisation, and low Personal Accomplishment were associated with poor quality of care [109, 110], but one study found that only Personal Accomplishment was related to better quality of care at the last shift [104]; Emotional Exhaustion and Cynicism predict low quality of care [54]; two articles reported that Emotional Exhaustion predicts poor nurse ratings of quality of care [82, 84]. A high burnout composite score predicted poor nurse-assessed quality of care [96]. In one instance, no associations were found between any of the burnout dimensions and quality of care [36].

Five studies considered aspects of patient safety: burnout was correlated with negative patient safety climate [111]. Emotional Exhaustion and Depersonalisation were both associated with negative patient safety grades and safety perceptions [112], and burnout fully mediated the relationship between depression and individual-level safety perceptions and work area/unit level safety perceptions [108]. Emotional Exhaustion mediated the relationship between workload and patient safety [51], and a higher composite burnout score was associated with lower patient safety ratings [113].

Regarding adverse events, high DEP and low Personal Accomplishment predicted a higher rate of adverse events [85], but in another study, only Emotional Exhaustion predicted adverse events [51]. When nurses were experiencing high levels of Emotional Exhaustion, they were less likely to report near misses and adverse events, and when they were experiencing high levels of Depersonalisation, they were less likely to report near misses [112].

All three MBI dimensions predicted medication errors in one study [109], but Van Bogaert et al. found that only high levels of Depersonalisation were associated with medication errors [104]. High scores in Emotional Exhaustion and Depersonalisation predicted infections [109]. Cimiotti et al. found that Emotional Exhaustion was associated with catheter-associated urinary tract infections and surgical site infections [114], while in another study, Depersonalisation was associated with nosocomial infections [104]. Lastly, patient falls were also explored, and Depersonalisation and low Personal Accomplishment were significant predictors in one study [109], while in a further study, only Depersonalisation was associated with patient falls [104]. There was no association between burnout and hospital-acquired pressure ulcers [20].

Considering patient experience, Vahey et al. concluded that higher Emotional Exhaustion and low Personal Accomplishment levels were associated with patient dissatisfaction [93], and Van Bogaert et al. found that Emotional Exhaustion was related to patient and family verbal abuse, and Depersonalisation was related to both patient and family verbal abuse and patient and family complaints [104].

In summary, evidence deriving from 17 studies points to a negative effect of burnout on quality of care, patient safety, adverse events, error reporting, medication error, infections, patient falls, patient dissatisfaction, and family complaints, but not on pressure ulcers.

### Individual characteristics

In total, 16 studies, which had examined work characteristics related to burnout, also considered the relationship between characteristics of the individual and burnout. Relationships were tested on demographic variables, including gender, age, and family status; on personality aspects; on work-life interference; and on professional attributes including length of experience and educational level. Because our focus on burnout is as a job-related phenomenon, we have not reported results of these studies into detail, but overall evidence on demographic and personality factors was inconclusive, and having family issues and high work-life interference was associated with different burnout dimensions. Being younger and not having a bachelor’s degree were found to be associated with a higher incidence of burnout.

## Discussion

This review aimed to identify research that had examined theorised relationships with burnout, in order to determine what is known (and not known) about the factors associated with burnout in nursing and to determine the extent to which studies have been underpinned by, and/or have supported or refuted, theories of burnout. We found that the associations hypothesised by Maslach’s theory between mismatches in areas of worklife and burnout were generally supported.

Research consistently found that adverse job characteristics—high workload, low staffing levels, long shifts, low control, low schedule flexibility, time pressure, high job and psychological demands, low task variety, role conflict, low autonomy, negative nurse-physician relationship, poor supervisor/leader support, poor leadership, negative team relationship, and job insecurity—were associated with burnout in nursing.

However few studies used all three MBI subscales in the way intended, and nine used different approaches to measuring burnout.

The field has been dominated by cross-sectional studies that seek to identify associations with one or two factors, rarely going beyond establishing correlation. Most studies were limited by their cross-sectional nature, the use of different or incorrectly applied burnout measures, the use of common methods (i.e. survey to capture both burnout and correlates), and omitted variables in the models. The 91 studies reviewed, while highlighting the importance of burnout as a feature affecting nurses and patient care, have generally lacked a theoretical approach, or identified mechanisms to test and develop a theory on the causes and consequences of burnout, but were limited in their testing of likely mechanisms due to cross-sectional and observational designs.

For example, 19 studies showed relationships between burnout and job satisfaction, missed care, and mental health. But while some studies treated these as *predictors* of burnout, others handled as *outcomes* of burnout. This highlights a further issue that characterises the burnout literature in nursing: the simultaneity bias, due to the cross-sectional nature of the evidence. The inability to establish a temporal link means limits the inference of causality [115]. Thus, a factor such as ‘missed care’ could lead to a growing sense of compromise and ‘crushed ideals’ in nurses [116], which causes burnout. Equally, it could be that job performance of nurses experiencing burnout is reduced, leading to increased levels of ‘missed care’. Both are plausible in relation to Maslach’s original theory of burnout, but research is insufficient to determine which is most likely, and thereby develop the theory.

To help address this, three areas of development within research are proposed. Future research adopting longitudinal designs that follow individuals over time would improve the potential to understand the direction of the relationships observed. Research using Maslach’s theory should use and report all three MBI dimensions; where only the Emotional Exhaustion subscale is used, this should be explicit and it should not be treated as being synonymous to burnout. Finally, to move our theoretical understanding of burnout forward, research needs to prioritise the use of empirical data on employee behaviours (such as absenteeism, turnover) rather than self-report intentions or predictions.

Addressing these gaps would provide better evidence of the nature of burnout in nursing, what causes it and its potential consequences, helping to develop evidence-based solutions and motivate work-place change. With better insight, health care organisations can set about reducing the negative consequences of having patient care provided by staff whose work has led them to become emotionally exhausted, detached, and less able to do the job, that is, burnout.

### Limitations

Our theoretical review of the literature aimed to summarise information from a large quantity of studies; this meant that we had to report studies without describing their context in the text and also without providing estimates (i.e. ORs and 95% CIs). In appraising studies, we did not apply a formal quality appraisal instrument, although we noted key omissions of important details. However, the results of the review serve to illustrate the variety of factors that may influence/result from burnout and demonstrate where information is missing. We did not consider personality and other individual variables when extracting data from studies. However, Maslach and Leiter recently reiterated that although some connections have been made between burnout and personality characteristics, the evidence firmly points towards work characteristics as the primary drivers of burnout [8].

While we used a reproducible search strategy searching MEDLINE, CINAHL, and PsycINFO, it is possible that there are studies indexed elsewhere and we did not identify them, and we did not include grey literature. It seems unlikely that these exist in sufficient quantity to substantively change our conclusions.

## Conclusion

Patterns identified across 91 studies consistently show that adverse job characteristics are associated with burnout in nursing. The potential consequences for staff and patients are severe. Maslach’s theory offers a plausible mechanism to explain the associations observed. However incomplete measurement of burnout and limited research on some relationships means that the causes and consequences of burnout cannot be reliably identified and distinguished, which makes it difficult to use the evidence to design interventions to reduce burnout.

## Supplementary information

**Additional file 1:** MEDLINE via OVID, CINAHL with full text via EBSCO, and PsycINFO via EBSCO.

**Additional file 2:** PRISMA-ScR Checklist.

**Additional file 3:** Studies’ settings, sample sizes, burnout and correlates measurement, and appraisal of quality.

## Data Availability

Not applicable

## Abbreviations

MBI: Maslach Burnout Inventory
CBI: Copenhagen Burnout Inventory
ProQoL5: Professional Quality of Life Measure
