# Supplementary material for: Burnout in nursing: a theoretical review
Source: Hum Resour Health. 2020 Jun 5;18:41. doi: 10.1186/s12960-020-00469-9 (PMC7273381; doi:10.1186/s12960-020-00469-9)
Supplement: Supplementary file 1 — Additional file 1: MEDLINE via OVID, CINAHL with full text via EBSCO, and PsycINFO via EBSCO. [file 12960_2020_469_MOESM1_ESM.docx]

| MEDLINE via OVID | 1 | burnout.mp. [mp=title, abstract, original title, name of substance word, subject heading word, keyword heading word, protocol supplementary concept word, rare disease supplementary concept word, unique identifier, synonyms] | 10400 |
| --- | --- | --- | --- |
|  | 2 | BURNOUT, PROFESSIONAL/ | 8470 |
|  | 3 | burn-out.mp. [mp=title, abstract, original title, name of substance word, subject heading word, keyword heading word, protocol supplementary concept word, rare disease supplementary concept word, unique identifier, synonyms] | 452 |
|  | 4 | MBI.mp. [mp=title, abstract, original title, name of substance word, subject heading word, keyword heading word, protocol supplementary concept word, rare disease supplementary concept word, unique identifier, synonyms] | 1148 |
|  | 5 | 1 or 2 or 3 or 4 | 11247 |
|  | 6 | exp Nursing Staff/ | 42053 |
|  | 7 | NURSES/ | 21058 |
|  | 8 | (nurse or nurses or nursing).tw. | 231102 |
|  | 9 | (RN or "RNs" or "RN's").tw. | 9153 |
|  | 10 | Nursing Administration Research/ | 2053 |
|  | 11 | Nursing Audit/ | 1867 |
|  | 12 | Models, Nursing/ | 9323 |
|  | 13 | 6 or 7 or 8 or 9 or 10 or 11 or 12 | 259501 |
|  | 14 | 5 and 13 | 3787 |
| CINAHL with full text via EBSCO | 1 | burnout | 10631 |
|  | 2 | (MH "Burnout, Professional+") | 8545 |
|  | 3 | burn-out | 317 |
|  | 4 | MBI | 776 |
|  | 5 | S1 OR S2 OR S3 OR S4 | 11292 |
|  | 6 | (MH "Staff Nurses") | 7677 |
|  | 7 | (MH "Nurses+") | 204040 |
|  | 8 | (nurse or nurses or nursing) | 816583 |
|  | 9 | (RN or RNs or RN's) | 19297 |
|  | 10 | (MH "Nursing Administration Research") | 635 |
|  | 11 | (MH "Nursing Audit") | 910 |
|  | 12 | (MH "Nursing Models, Theoretical") | 4483 |
|  | 13 | S6 OR S7 OR S8 OR S9 OR S10 OR S11 OR S12 | 823083 |
|  | 14 | S5 AND S13 | 4632 |
| PsycINFO via EBSCO | 1 | Burnout | 13262 |
|  | 2 | DE "Occupational Stress" | 20264 |
|  | 3 | burn-out | 509 |
|  | 4 | MBI | 1402 |
|  | 5 | S1 OR S2 OR S3 OR S4 | 25539 |
|  | 6 | DE "Nursing" OR DE "Nurses" | 40388 |
|  | 7 | (nurse or nurses or nursing) | 153297 |
|  | 8 | RN or RNs or RN's | 2064 |
|  | 9 | S6 OR S7 OR S8 | 153898 |
|  | 10 | S5 AND S9 | 3829 |
