# Supplementary material for: Burnout in nursing: a theoretical review
Source: Hum Resour Health. 2020 Jun 5;18:41. doi: 10.1186/s12960-020-00469-9 (PMC7273381; doi:10.1186/s12960-020-00469-9)
Supplement: Supplementary file 3 — Additional file 3: Studies’ settings, sample sizes, burnout and correlates measurement, and appraisal of quality. [file 12960_2020_469_MOESM3_ESM.docx]

| Author, Year | Country | Setting | Sample Size | Staff group | Measure of burnout | Variables (measures) | Generalisable sample * | Common methods variance** | Clustering*** | Statistical adjustment**** |
| --- | --- | --- | --- | --- | --- | --- | --- | --- | --- | --- |
| Adali et al, 2003 | Greece | Hospital – specialty  (Psychiatry) | 199 nurses, 5 hospitals | Nursing personnel in psychiatric hospitals | MBI | **Predictors (Moos Work Environment Scale):**   - Co-worker cohesion - Supervisor support - Autonomy - Task orientation - Work pressure   Clarity | N | Y | N | N |
| Anagnostopoulos and Niakas, 2010 | Greece | Hospital - all wards | 487 RNs, 4 hospitals | RNs with direct patient care and full time employment | MBI | **Outcomes:**  Short-term sickness absence (HR records) | N | N | N | N |
| Aiken et al, 2008 | US | Hospital - all wards | 10184 RNs, 168 hospitals | RNs | MBI (EE) | **Predictors:**   - Patient Care environment (PES-NWI)   Nurse staffing levels: mean patients/nurse (study questionnaire) | Y | Y | Y | Y |
| Aiken and Sloane, 1997 | US | Hospital – specialty  (AIDS) | 820 RNs, 20 hospitals | RNs | MBI (EE) | **Predictors:**  Type of hospital: presence/absence of specialised AIDS units (administrative data) | Y | N | N | Y |
| Aiken et al, 2002 | US | Hospital - all wards | 10184 RNs, 210 hospitals | RNs | MBI (EE) | **Predictors:**  Nurse staffing levels: patient-to-nurse ratio (nurse survey) | Y | Y | Y | Y |
| Akman et al, 2016 | Turkey | Hospital – specialty  (Paediatric) | 165 RNs, 10 clinics, 4 hospitals | Paediatric RNs | MBI (composite in regression) | **Predictors:**   - Nurse staffing levels: number of patients taken care of during daytime (socio-demographic form)   Job satisfaction (Minnesota job satisfaction scale) | N | Y | N | N |
| Andela et al, 2016 | Country not reported | Hospital - all wards | 445 healthcare workers, 1 hospital | RNs, health care assistants | MBI (PA dropped because NS) | **Predictors:**   - Patients’ and relatives’ requirements (Work Stress Inventory for Nurses in Oncology – WSINO) - Patients’ suffering (WSINO) - Workload (WSINO) - Team collaboration problems (WSINO)   Emotional dissonance (authors developed scale) | N | Y | N | Y |
| Anwar and Elareed, 2017 | Egypt | Hospital - all wards | 286 RNs, 1 hospital | RNs | MBI-HSS (composite in regression) | **Predictors (study questionnaire):**   - Number of shifts - Type of violence (from staff – verbal or emotional)   Shift timing | N | Y | N | N |
| Bagheri Hosseinabadi et al, 2019 | Iran | Hospital - all wards | 682 nurses, 4 teaching hospitals | Nurses | MBI-GS (both separate scales and composite measure) | **Predictors:**   - Age (demographic questionnaire) - Gender (demographic questionnaire) - Marital status (demographic questionnaire) - Length of experience (demographic questionnaire) - Irregular shifts (demographic questionnaire) - Psychological demand (Persian Job Content Questionnaire)   Workplace support (Persian Job Content Questionnaire) | N | Y | N | Y |
| Basar and Basim, 2016 | Turkey | Hospital - all wards | 456 RNs, 6 hospitals | RNs | Malach-Pines Burnout measure scale (BMS-10) | **Outcomes:**   - Intention to quit (Walsh scale) - Perceptions of organizational politics (Hochwarter scale)   Neglect of work (Rusbult scale) | N | Y | N | N |
| Basinska and Wilczek-Ruzyczka, 2013 | Poland | Hospital - general | 263 RNs | RNs | MBI | **Predictors:**   - Job demands (Effort-Reward Imbalance questionnaire)   Rewards: esteem, job promotion and salary, job security (Effort-Reward Imbalance questionnaire) | N | Y | N | N |
| Boamah and Laschinger, 2016 | Canada | All settings | 215 RNs | Newly graduated nurses (RNs) | MBI (EE and CYN) | **Predictors**:   - Areas of work-life (Areas of Worklife Scale) - Work-life interference (Work Interference with Personal Life scale)   **Outcomes**:  Intention to leave (Turnover Intentions scale) | N | Y | N | N |
| Boamah et al, 2017  *Time-lagged study* | Canada | All settings | 1021 RNs at time 1; 406 at time 2 | Newly graduated nurses (RNs) | MBI (EE and CYN) | **Predictors:**   - Authentic leadership (Authentic Leadership Questionnaire) - Structural empowerment (Conditions of Work Effectiveness-II) - Short staffing (study questionnaire) - Work-life interference (Work Interference with Personal Life scale)   **Outcomes:**   - Job satisfaction (Michigan Assessment of Organisations questionnaire)   Nurse-assessed quality of care (study questionnaire) | N | Y | N | N |
| Bobbio et al, 2012 | Italy | Hospital - all wards | 273 RNs, 1 hospital | RNs | MBI-GS (composite) | **Predictors (study questionnaire):**   - Empowering leadership - Trust in the leader   Trust in organization | N | Y | N | N |
| Bourbonnais et al, 1998  *Longitudinal* | Canada | Hospital - all wards | 1891 RNs, 6 hospitals | RNs | MBI (EE) | **Predictors (study questionnaire)**:   - Psychological demands   Decision latitude | Y | Y | N | Y |
| Cañadas-De la Fuente et al, 2015 | Spain | Primary Care and Community | 676 RNs | RNs | MBI | **Predictors:**   - Level of healthcare: hospital, primary (study questionnaire) - Healthcare service area (study questionnaire) - Job shift (study questionnaire) - On call requirement (study questionnaire) - Administrative tasks (study questionnaire) - Seniority in current job and profession (study questionnaire) - Neuroticism (NEO-FFI) - Agreeableness (NEO-FFI)   Extraversion (NEO-FFI) | Y | Y | N | Y |
| Cao and Naruse, 2019 | Japan | Primary Care and Community | 93 nurses | Licenced nurses working for agency | Japanese Burnout Inventory (EE, DEP) | **Predictors:**   - Time pressure (study questionnaire)   Relational Coordination with nurse managers (Relational Coordination Scale) | N | Y | N | Y |
| Cimiotti et al, 2012 | US | Hospital - all wards | 7076 RNs, 161 hospitals | RNs | MBI (EE) | **Outcomes:**   - Catheter-associated urinary tract infections (PHC4)   Surgical site infections (PHC4) | Y | N | N | Y |
| Colindres et al, 2018 | Ecuador | Hospital - all wards | 333 nurses from 4 hospitals | Nurses (auxiliary, general or specialized) | Copenhagen Burnout Inventory (CBI) | **Predictors:**   - Effort/reward imbalance (ERI questionnaire)   Adherence to infection prevention and control (Johns Hopkins University School of Hygiene and Public Health Safety Climate Questionnaire) | N | Y | N | N |
| Dall’Ora et al, 2015 | RN4CAST (12 EU countries) | Hospital - general | 31627 RNs, 2170 units, 488 hospitals | RNs | MBI | **Predictor:**  Shift length (study questionnaire) | Y | Y | Y | Y |
| Dhaini et al, 2018 | Switzerland | Hospital - all wards | 1833 RNs, 23 hospitals, 124 units, | RNs | MBI (EE) | **Predictors:**   - Work schedule flexibility (study questionnaire) - Perceived nurse manager ability (PES-NWI) - Work family conflict (Work-Family Conflict scale) - Familial status (study questionnaire)   Workload: number of patients to care for (study questionnaire) | Y | Y | Y | Y |
| Dutra et al, 2018 | Brazil | Hospital - all wards | 3 hospitals, 450 nurses | RNs and nurse technicians | MBI-HSS (only EE) | **Outcomes:**   - Job dissatisfaction (Safety Attitudes Questionnaire)   Intent to leave (Study questionnaire) | N | Y | N | Y |
| Estryn-Béhar et al, 2007 | NEXT (10 EU countries) | Hospital - all wards | 28561 RNs | RNs | CBI | **Outcome:**  Intention to leave (Study questionnaire) | Y | Y | Y | Y |
| Faller et al, 2011 | US | Hospital - all wards | 976 RNs | Travel RNs (temporary nurses) | CBI | **Predictors (Study questionnaire):**   - Age - Gender - Race - Married - Children at home - Education level - Years of experience as RN - Years of experience as traveller - Hours worked per week - Staffing levels: number of patients per shift - Ward type - Magnet   Location of facility | N | Y | N | N |
| Firth and Britton, 1989  *Longitudinal* | UK | Hospital - general | 106 nurses | Qualified nursing staff | MBI | **Outcomes (Study questionnaire):**   - Episodes of absence   Turnover | N | Y | N | N |
| Flynn et al, 2009 | US | Primary Care and Community | 1,015 RNs | RNs in haemodialysis settings | MBI (EE) | **Predictors:**   - RN staffing levels: patient-to-nurse ratio (study questionnaire) - Workload (Individual Workload Perception Scale) - Practice environments (PES-NWI-R) - Impaired nursing care processes (study questionnaire)   **Outcomes**:   - Intention to leave current position (Study questionnaire)   Intention to leave current employer (Study questionnaire) | Y | Y | N | N |
| Galletta et al, 2016 | Italy | Hospital - all wards | 307 RNs, 1 hospital, 24 units | RNs | 16-items MBI | **Predictors:**   - Organisational empowerment (Conditions of Work Effectiveness Questionnaire-II) - Workload (Areas of Worklife Scale – AWS) - Job control (AWS)   Team quality (modified ICU questionnaire) | N | Y | N | Y |
| Gandi et al, 2011 | Nigeria | Hospital - all wards | 2245 RNs | RNs | MBI | **Predictors:**   - Workload (Job autonomy questionnaire) - Job control (Inventory of Feelings of Motivation and Demotivation) - Work-home interference (study questionnaire)   Home-work interference (study questionnaire) | N | Y | N | N |
| Garcia-Sierra et al, 2016 | Spain | Hospital - general | 100 RNs, 2 hospitals | RNs | MBI (EE and CYN) | **Predictors**:   - Demands (Job Content Questionnaire)   Support (Job Content Questionnaire) | N | Y | N | Y |
| Garrett and McDaniel, 2001 | US | Hospital - all wards | 77 RNs, 1 hospital, 5 units | RNs | MBI | P**redictors:**   - Environmental uncertainty (Perceived environmental uncertainty Scale)   Social climate: Supervisor Support and Peer cohesion (Work Environment Scale) | N | Y | N | N |
| Giorgi et al, 2018 | Italy | Hospital - all wards | 315 RNs, 39 units, 7 hospitals | RNs | CBI | **Predictors**:   - Quality of sleep (Pittsburgh Sleep Quality Index)   **Outcomes:**   - Quality of Sleep   Job performance (Job performance scale: Task performance and contextual performance) | Y | Y | N | Y |
| Greco et al, 2006 | Canada | Hospital - all wards | 322 RNs | RNs | MBI (EE) | **Relationships:**   - Leader empowering behaviours (Leader Empowering Behaviour Scale) - Structural empowerment (Conditions of Work Effectiveness Questionnaire–II)   Areas of work-life (Areas of Worklife Scale) | N | Y | N | N |
| Greenglass, 2001 | Canada | Hospital - all wards | 1363 nurses, 11 nursing units | Nurses employed in hospitals undergoing restructuring | MBI-GS | **Predictor:**   - Workload (study questionnaire)   **Outcome:**  Somatization (Hopkins Symptom Checklist) | Y | Y | N | N |
| Halbesleben et al, 2008 | US | Hospital - all wards | 148 Nurses, 1 hospital | RNs, Licenced Practice Nurses, Nurse Practitioners | MBI (EE and DEP) | **Outcomes (AHRQ Patient Safety Culture Survey)**:   - Patient safety grade - Safety perceptions - Event reports   Near-miss frequency reporting | N | Y | N | Y |
| Hanrahan et al, 2010 | US | Hospital – specialty  (Psychiatry) | 353 RNs, 67 hospitals | Psychiatric RNs | MBI | **Predictors:**   - Practice Environment (PES-NWI) - Nurse Participation in Hospital Affairs (PES-NWI) - Foundations for Quality of Care (PES-NWI) - Manager Skill and Leadership (PES-NWI) - Nurse-Physician Relationship (PES-NWI)   Nurse Staffing: Patient to Nurse Ratio (study questionnaire) | Y | Y | N | Y |
| Hunsaker et al, 2015 | US | Hospital - critical care/ED | 278 RNs | Emergency Department RNs | ProQol 5 (Burnout subscale) | **Predictors (study questionnaire)**:   - Level of education - Years in profession - Hours of work per week - Shift length - Years as an ED nurse   Manager support | N | Y | N | Y |
| Ilhan et al, 2008 | Turkey | Hospital - all wards | 418 RNs, 1 hospital | RNs | MBI | **Predictors (study questionnaire):**   - Number of years in nursing - Weekly work duration - Shift-working - Workload - Perceived health - Personal difficulties   Financial difficulties | N | Y | N | N |
| Jansen et al, 1996 | The Netherlands | Primary Care and Community | 441 nurses | Community RNs and auxiliaries | MBI-NL (Dutch version) | **Predictors (Algera questionnaire):**   - Time pressure - Autonomy - Task clarity - Skill variety - Growth at work - Feedback - Task significance - Active approach - Passive approach - Seeking social support - Support received from head nurse   Support received from peers | N | Y | N | Y |
| Janssen et al, 1999 | The Netherlands | Hospital - all wards | 156 RNs | RNs, head nurses, nurse aids | MBI (EE) | **Predictors (Study questionnaire):**   - Quality of job content - Mental work overload - Social support - Unmet career expectations   **Outcomes:**   - Intrinsic work motivation (Warr scale)   Turnover iteration (study questionnaire) | N | Y | N | N |
| Johnson et al, 2017 | UK | Hospital - all wards | 232 nursing staff, 3 hospitals | RNs, healthcare assistants | MBI (EE and DEP) | **Predictors:**   - Depressive symptoms (Depression, Anxiety and Stress scale)   **Outcomes (AHRQ Hospital Survey on Patient Safety Culture):**   - Individual level safety perception   Work area/unit level safety perceptions | N | Y | Y | Y |
| Kanai-Pak et al, 2008 | Japan | Hospital - all wards | 5956 staff nurses, 302 units, 19 hospitals | Staff nurses | MBI (EE) | **Predictors**:   - Inexperienced nurses (study questionnaire) - Staffing-resources adequacy (NWI-R)   Nurse physician relations (NWI-R) | Y | Y | N | N |
| Kanste et al, 2007 | Finland | All settings | 601 RNs and nurse managers | RNs, specialist nurses, nurse managers | MBI-HSS | **Predictors (Multifactor Leadership Questionnaire):**   - Rewarding transformational leadership - Active management-by-exception   Passive laissez-faire leadership | N | Y | N | Y |
| Khamisa et al, 2016 | South Africa | Hospital - all wards | 277 nurses, 4 hospitals | Nurses | MBI-HSS (composite score in regression) | **Predictors:**   - Work related stress parameters: patient care, staff issues, job demands, overtime (Nursing Stress Inventory)   **Outcomes:**   - General health (General Health Questionnaire)   Job satisfaction: Pay, promotion, supervision, fringe benefits, operating conditions, contingent rewards, co-workers, nature of work, communication (Job Satisfaction Survey) | N | Y | N | N |
| Kitaoka-Higashiguchi, 2005 | Japan | Hospital - all wards | 238 nurses, 1 hospital | Nurses | MBI-GS | **Predictors:**   - Workload demand (Job Content Questionnaire) - Decision authority (Job Content Questionnaire) - Role conflict (NIOSH questionnaire) - Interpersonal conflict (NIOSH questionnaire)   Supervisor support and co-worker support (NIOSH) | N | Y | N | N |
| Klopper et al, 2012 | South Africa | Hospital - critical care/ED | 935 nurses, 42 hospitals | Critical care nurses | MBI | **Predictors:**   - Practice Environment (PES-NWI)   Job satisfaction (Study questionnaire) | Y | Y | N | N |
| Konstatinou et al, 2018 | Greece | Primary Care and Community | 78 nurses, 4 clinics | Mental health nurses | MBI | **Predictors:**   - Role conflict (Role Conflict Scale) - Training (Study questionnaire) - Pay (Study questionnaire) - Serious family issue (Study questionnaire)   Workload (Study questionnaire) | N | Y | N | N |
| Kowalski et al, 2010 | Germany | Hospital - all wards | 959 nurses, 4 hospitals | Nurses | MBI-GS (EE) | **Predictors:**   - Workload (Intensity of labour scale) - Decision latitude (Intensity of labour scale)   Social capital in the hospitals (Social capital in organisations) | Y | Y | N | Y |
| Laeeque et al, 2018 | Pakistan | Hospital - all wards | 216 nurses, 4 hospitals | Nurses | CBI | **Predictor:**   - Patient violence (Study questionnaire)   **Outcome:**  Intention to leave (Ganesan and Weitz scale) | N | Y | N | N |
| Laschinger et al, 2001 | Canada | Hospital - general | 3016 nurses, 135 hospitals | Staff nurses | MBI-HSS (EE) | **Predictors:**   - Organizational characteristics: autonomy, control, collaboration (NWI) - Organizational trust (Interpersonal Trust at Work Scale)   **Outcomes:**   - Job satisfaction (Study questionnaire)   Nurse-assessed quality (Study questionnaire) | Y | Y | N | N |
| Laschinger and Leiter, 2006 | Canada | Hospital - all wards | 8597 nurses | Hospital-based nurses | MBI | **Predictors (NWI-PES) :**   - Strong leadership - Policy involvement - RN/MD Collaboration - Staffing adequacy - Nursing of care   **Outcome:**  Adverse events (Study questionnaire) | Y | Y | N | N |
| Laschinger et al, 2009 | Canada | Hospital - all wards | 612 nurses, 5 hospitals | Nurses | MBI (EE and CYN) | **Outcomes:**   - Job satisfaction (Study questionnaire) - Organizational commitment (Affective Commitment Scale)   Turnover Intentions (Turnover Intentions measure) | Y | Y | N | N |
| Laschinger, 2012 | Canada | Hospital - all wards | 342 nurses | Newly graduate nurses | MBI (EE and CYN) | **Outcomes:**   - Job satisfaction (Satisfaction Scale) - Intention to leave the job (Turnover Intent scale) - Career satisfaction (Satisfaction Scale)   Intention to leave nursing (Turnover Intent scale) | N | Y | N | N |
| Laschinger et al, 2012 | Canada | Hospital - all wards | 342 nurses | Newly graduate nurses | MBI (EE) | **Predictors:**   - Authentic leadership (Authentic Leadership Questionnaire) - Workplace bullying (Negative Acts questionnaire)   **Outcomes:**   - Job satisfaction (Satisfaction Scale)   Turnover intentions (Turnover Intent scale) | N | Y | N | N |
| Laschinger et al, 2015 | Canada | Hospital - all wards | 1009 nurses | Newly graduate nurses | MBI | **Relationships:**   - Authentic leadership (Authentic Leadership Questionnaire) - Areas of work-life (Areas of Worklife Scale) - Occupational coping self-efficacy (Occupational Coping self-efficacy scale)   Mental health (General Health Questionnaire) | Y | Y | N | N |
| Laschinger and Read, 2016 | Canada | All settings | 993 nurses | New graduate nurses | MBI (EE) | **Relationships:**   - Authentic leadership (Authentic Leadership Questionnaire) - Areas of work life (Areas of Worklife Scale) - Civility norms (Civility Norms Questionnaire)   Coworker incivility (Straightforward Workplace Incivility Scale) | Y | Y | N | N |
| Lee et al, 2019 | Taiwan | Hospital - all wards | 946 nurses | Nurses – analysis broken down by seniority | MBI‐HSS (EE) | **Predictor:**   - Authentic leadership (Authentic Leadership Questionnaire)   **Outcome:**  Intention to leave (Intent-To-Leave job questionnaire) | Y | Y | N | N |
| Leineweber et al, 2014 | Sweden | Hospital - general | 8620 RNs, 53 hospitals, 369 departments, | RNs | MBI | **Predictors:**   - Work-family conflict (Study questionnaire) - Staff adequacy (PES-NWI) - Leadership and support for nurses (PES-NWI)   Nurse-physician relationship (PES-NWI) | Y | Y | Y | Y |
| Leiter and Laschinger, 2006 | Canada | Hospital - all wards | 8597 nurses | Nurses | MBI | **Relationships (PES-NWI):**   - Leadership - Policy involvement - RN/MD collaboration - Staffing adequacy   Nursing model of care | Y | Y | N | N |
| Leiter and Masclach, 2009 | Canada | All settings | 667 nurses | RNs, Licenced practical nurses, clinical nurse specialists, Clinical Nurse Educators, Nurse Practitioners | MBI-GS | **Predictors (Areas of Work Life scale):**   - Control - Workload - Community - Fairness - Reward - Value congruence   **Outcome:**  Turnover intentions (Turnover Intentions scale) | Y | Y | N | N |
| Levert et al, 2000 | South Africa | Hospital - specialty | 94 nurses, 4 hospitals | Psychiatric nurses | MBI | **Predictors:**   - Workload (Workload and Lack of Collegial Support scale) - Collegial support (Workload and Lack of Collegial Support scale) - Role conflict (Role Conflict and Role Ambiguity scale) - Role Ambiguity (Role Conflict and Role Ambiguity scale)   **Mediator:**  Sense of coherence (Orientation to Life Questionnaire) | N | Y | N | N |
| Li et al, 2013 | RN4Cast (11 EU countries) | Hospital - general | 23,446 RNs, 352 hospitals, 2087 units | RNs | MBI | **Predictors (PES-NWI):**   - Managerial support for nursing - Doctor-nurse collegial relations   Promotion of care quality | Y | Y | Y | Y |
| Liu and Aungsuroch, 2018 | China | Hospital - all wards | 510 nurses, 4 hospitals | Nurses | MBI-HSS (composite score) | **Relationships:**   - Work environment (Chinese-PES) - Patient-to-nurse ratio (Nurse Staffing Form) - Job satisfaction (Chinese Nurse Job Satisfaction Scale) - Nurse-assessed quality nursing care (Chinese Nurse Assessed Quality of Nursing Care Scale)   Intention to leave (Chinese Anticipated Turnover Scale) | N | Y | N | N |
| Liu et al, 2018 | China | Hospital - general | 1542 nurses, 23 hospitals, 111 units | Nurses | MBI-HSS (EE) | **Relationships:**   - Work environment (PES-NWI) - Workload (patient-nurse-ratio and non-professional tasks) - Patient safety (Study questionnaire)   Adverse events (Study questionnaire) | Y | Y | N | N |
| Lu et al, 2015 | China | Hospital - all wards | 856 nurses | Nurses | MBI (EE) | **Predictor:**  Patient-nurse ratio (Study questionnaire) | N | Y | N | Y |
| Madathil et al, 2014 | US | Hospital – specialty  (Psychiatry) | 89 nurses, 2 hospitals | RNs and others not specified | MBI-HSS | **Predictors:**   - Workload (Study questionnaire) - Autonomy (NWI-R)   Transformational leadership (Multifactor Leadership) | N | Y | N | Y |
| Marques-Pinto et al, 2018 | Portugal | Hospital - general | 2235 nurses, 31 hospitals | Nurses | MBI (EE) | **Predictors:**   - Job demands (PES-NWI) - Nurses’ participation in hospital affairs (PES-NWI)   **Outcomes**:  Intention to leave (Study Questionnaire) | Y | Y | N | N |
| Moloney et al, 2018 | New Zealand | All settings | 2876 nurses | Nurses | Malach-Pines Burnout measure scale (BMS-10) | **Relationships:**   - Job demands: quantitative demands, Emotional demands-hindrances, Emotional demands-challenges (Job-demands scale) - Personal demands: Work-life interference (Work-life interference scale) - Job resources; Supervisor support and Colleague support (Supervisor and Colleague Support scale), Organizational support (Organizational Support Scale), Autonomy (Work Design Questionnaire), Professional development (Professional Development scale) - Personal resources: Psychological capital/self-efficacy (PsyCap questionnaire), Value congruence (Retention model) - Intention to leave organization (Retention survey)   Intention to leave profession (Retention survey) | Y | Y | N | N |
| Nantsupawat et al, 2016 | Thailand | Hospital – specialty  (Community) | 2084 RNs, 94 hospitals | RNs | MBI | **Outcomes (Study questionnaire):**   - Quality of care - Patient falls - Medication errors   Infections | Y | Y | Y | Y |
| Nantsupawat et al, 2017 | Thailand | Hospital - all wards | 1351 nurses, 43 units 5 hospitals | Inpatient nurses | MBI (EE) | **Predictor:**  Nurse work environment (PES-NWI) | Y | Y | Y | Y |
| Parker and Kulik, 1995 | US | Hospital - all wards | 73 RNs | RNs providing direct patient care | MBI | **Predictors:**   - Job stress (Nursing Stress Scale) - Social work-related support (Social Support Scale)   **Outcomes:**   - Self-rated performance (Nurse questionnaire) - Supervisor-rated performance (Clinical Services Director questionnaire) - Reported Absenteeism (Mental) (Nurse questionnaire) - Reported Absenteeism (Physical) (Nurse questionnaire) - Sick leave (HR records)   Intention to quit (Nurse questionnaire) | N | Y | N | Y |
| Poghosyan et al, 2010 | US, Canada, UK Germany, New Zealand and Japan | Hospital - all wards | 52709 nurses, 616 hospitals | Nurses | MBI | **Outcome:**  Nurse-rated quality of care (Study questionnaire) | Y | Y | Y | Y |
| Poncet et al, 2007 | France | Hospital - critical care/ED | 2392 nurses, 278 units | Registered nurses, nurse assistants, head nurses | MBI (composite score) | **Predictors (Study questionnaire):**   - Able to schedule days off according to personal wishes - Participates in a research group - Conflicts with patients - Relationship with head nurses - Relationship with physicians - Caring for a dying patient   Number of decisions to forego life-sustaining therapies last week | Y | Y | N | Y |
| Rouxel, 2016 | France | Primary Care and Community | 371 nurses, 79 geriatric care centres | Geriatric care nurses | MBI (EE, DEP) | **Relationships:**   - Affectivity (Positive and Negative Affect scale) - Occupational group (nurses vs auxiliary nurses) - Perceived display rules (Emotion Work Requirements scale) - Job demands (Job-content questionnaire) - Job control (Job-Content questionnaire)   Job satisfaction (Work Design questionnaire) | Y | Y | N | N |
| Rudman and Gustavsson, 2011  *Prospective longitudinal* | Sweden | All settings | 997 RNs | Newly graduate registered nurses | Scale adapted from Job demands-resources model of burnout | **Predictors-all measured by study questionnaire unless indicated:**   - Age - Gender - Children - Country of birth - Previous training as nurse assistant - Previous work experience in healthcare - Performance-based self-esteem (PBSE scale) - Affectivity (Personality Inventory) - Self-rated health - Eating habits - Smoking - Alcohol consumption (Alcohol Use Disorders Identification Test) - Depressive mood - Depressive episode (Major Depression Inventory) - Musculoskeletal tension and pain - Back pain - Neck and shoulder pain - Burnout symptoms during studies - Present stressors - Importance of nursing studies - Overall educational outcome   Satisfaction with induction | Y | Y | N | Y |
| Shamian et al, 2002 | Canada | Hospital - critical care/ED | 6188 RNs, 160 hospitals | RNs | MBI (EE) | **Predictors:**   - Merged hospital (Study questionnaire) - Hospital type (Study questionnaire) - Percentages of nurses full-time (Study questionnaire) - Nurse autonomy (NWI-R) - Control over practice setting (NWI-R) - Nurse-physician relations (NWI-R)   Effort and reward imbalance (ERI score) | Y | Y | N | Y |
| Shao et al, 2018 | China | Hospital - all wards | 19,184 RNs | RNs | C-MBI (EE, DEP) | **Predictor:**   - Work environment (Chinese Nursing Work Environment scale)   **Moderator:**  Value congruence (Perceived fit scale) | Y | Y | Y | Y |
| Smith Lewis and Cunnigham, 2016 | US | Hospital - all wards | 120 nurses | Nurses | MBI-GS (composite score) | **Predictor:**   - Transformational leadership (Rafferty and Griffin scale)   **Mediators:** Areas of work life: Manageable workload, control, reward, community, fairness, values (Areas of Worklife Scale) | N | Y | N | Y |
| Stimpfel et al, 2012 | US | Hospital - general | 22,275 RNs, 577 hospitals | RNs | MBI (EE) | **Predictor:** Shift length (Study questionnaire) | Y | Y | Y | Y |
| Stone et al, 2007 | US | Hospital - all wards | 2047 nurses, 13 hospitals | Mainly RNs (unclear which other groups) | MBI | **Predictors (Perception of Nurse Work Environment scale):**   - Professional practice - Nurse/physician collaboration - Low nurse management - Positive scheduling climate - Low opportunity for advancement   Low unit decision-making | Y | Y | Y | Y |
| Teng et al, 2010 | Taiwan | Hospital - general | 458 nurses, 90 units, 2 hospitals | Nurses | MBI (composite score in regression) | **Outcome:**  Patient safety (Study questionnaire) | N | Y | N | Y |
| Thompson, 2014  *Longitudinal* | US | Hospital - general | 2011: 326,750 nurses, 12,915 units, 677 hospitals 927 2012: 327,396 nurses, 18,874 units, 927 hospitals | RNs | Scale developed for study (2 questions) | **Predictors (Practice Environment Scale):**   - Nurse manager leadership - RN and medical doctor collaboration - Policy involvement - Staffing adequacy - Nursing model of care   **Mediator:**   - Missed care (NDNQI Survey)   **Outcomes:**  Hospital-acquired pressure ulcer prevalence rate | Y | Y | N | Y |
| Tourigny et al, 2010 | Japan and China | All settings | 789 nurses (239 Japan, 550 China) | Nurses | MBI | **Relationships:**   - Depression (Centre for Epidemiological Studies Depression Survey) - Job satisfaction (Stephen Kerr scale)   Absenteeism (Study questionnaire) | Y | Y | Y | Y |
| Vahey et al, 2004 | US | Hospital – specialty (AIDS) | 820 nurses, 621 patients, 40 units, 20 hospitals | Staff nurses (RN’s and LPN’s) | MBI | **Predictor:**   - Work environment (NWI-R)   **Outcomes:**  Patient satisfaction (La Monica-Oberst Patient Satisfaction Scale) | Y | Y | Y | Y |
| Van Bogaert et al, 2009 | Belgium | Hospital - general | 401 nurses, 31 units, 2 hospitals | Staff nurses | MBI | **Relationships:**   - Nurse-physician relationship (NWI-R) - Nurse management at the unit level (NWI-R) - Hospital management & organizational support (NWI-R) - Nurse-assessed quality of care (Study questionnaire)   Job outcomes: job satisfaction, intention to stay in current hospital, intention to stay in nursing, and apply for a job in the last year (Study questionnaire) | N | Y | N | N |
| Van Bogaert et al, 2013 | Belgium | Hospital - all wards | 1201 RNs, 116 units, 8 hospitals | RNs | MBI | **Relationships:**   - Nurse-physician relationship (NWI-R) - Nurse management at the unit level (NWI-R) - Hospital management & organizational support (NWI-R) - Workload (Intensity of Labour scale) - Decision latitude (Decision latitude scale) - Social capital (Study questionnaire) - Nurse-assessed quality of care (Study questionnaire)   Job outcomes: job satisfaction, intention to stay in current hospital, intention to stay in nursing, and apply for a job in the last year (Study questionnaire) | Y | Y | N | N |
| Van Bogaert et al, 2014 | Belgium | Hospital - all wards | 1108 RNs, 96 units, 2 hospitals | RNs | MBI | **Predictors:**   - Nurse-physician relationship (NWI-R) - Nurse management at the unit level (NWI-R) - Hospital management & organizational support (NWI-R) - Workload (Intensity of Labour scale) - Decision latitude (Decision latitude scale)   **Outcomes (Study questionnaire):**   - Job satisfaction - Job outcomes: job satisfaction, intention to stay in nursing (Study questionnaire) - Quality of care of the current unit - Quality of care at last shift - Quality of care in hospital the last year - Patient and family complaints - Patient and family verbal abuse - Patient falls - Nosocomial infections   Medication errors | Y | Y | N | N |
| Vidotti et al, 2018 | Brazil | Hospital - all wards | 502 nurses, 1 hospital | Nurses | MBI-HSS | **Predictors:**   - Psychological demands (Demand-Control Support Questionnaire) - Work control (Demand-Control Support Questionnaire) - Social support received at work (Demand-Control Support) - Shift type (Demand-Control Support Questionnaire) - Dissatisfaction with sleep (Demand-Control Support Questionnaire) - Children (Demand-Control Support Questionnaire) - Number of years at the institution (Demand-Control Support Questionnaire)   Job type (Demand-Control Support Questionnaire) | N | Y | N | Y |
| Wisetborisut et al, 2014 | Thailand | Hospital - all wards | 2772 nurses, 1 hospital | Health-care workers, nurses, nurse aid, other | MBI (burnout= 1 high score in at least one subscale) | **Predictors (Study questionnaire):**   - Total number of shifts per month - Number of night shifts per month - Number of years working shifts - Days off per month   Sleeping time (hours per day) | N | Y | N | Y |
| Zarei et al, 2016 | Iran | Hospital - all wards | 250 nurses, 4 hospitals | RNs | MBI | **Outcome:**  Patient safety climate (study sample) | N | Y | N | N |
| Zhang et al, 2014 | China | Hospital - all wards | 9698 nurses, 181 hospitals | Nurses | MBI | **Predictors:**   - Work environment (PES-NWI) - Employment status (Study questionnaire)   Education level (Study questionnaire) | Y | Y | N | Y |
| Zhou et al, 2015 | China | Hospital - general | 1100 nurses 20 hospitals | Nurses | MBI (EE) | **Predictors:**   - Hospital-level nurse staffing (Study questionnaire) - Nurse-rated working environment (PES-NWI)   Nursing actual working time (Study questionnaire) | Y | Y | N | Y |
| * A multisite study with more than 500 participants ;  ** Burnout and correlates assessed with the same survey; it arises when there is shared (common) variance because of the common method rather than a true (causal) association between variables)  *** Nurses nested in wards, wards nested in hospitals)  **** The association between burnout and correlates has been adjusted for potentially influencing variables. | | | | | | | | | | |
